# Nigral ATP13A2 depletion induces Parkinson’s disease-related neurodegeneration in a pilot study in non-human primates

**DOI:** 10.1038/s41531-024-00757-4

**Published:** 2024-08-01

**Authors:** Joanna Sikora, Sandra Dovero, Rémi Kinet, Marie-Laure Arotcarena, Sylvain Bohic, Erwan Bezard, Pierre-Olivier Fernagut, Benjamin Dehay

**Affiliations:** 1 Univ. Bordeaux, CNRS, IMN, Bordeaux, France; 2 Univ. De Poitiers, INSERM, LNEC, Poitiers, France; 3 Univ. Grenoble Alpes, Synchrotron Radiation for Biomedicine (STROBE), Grenoble, France

**Keywords:** Parkinson's disease, Neurodegeneration, Parkinson's disease

## Abstract

Lysosomal impairment is strongly implicated in Parkinson’s disease (PD). Among the several PD-linked genes, the *ATP13A2* gene, associated with the PARK9 locus, encodes a transmembrane lysosomal P5-type ATPase. Mutations in the *ATP13A2* gene were primarily identified as the cause of Kufor-Rakeb syndrome (KRS), a juvenile-onset form of PD. Subsequently, an increasing list of several mutations has been described. These mutations result in truncation of the ATP13A2 protein, leading to a loss of function but surprisingly causing heterogeneity and variability in the clinical symptoms associated with different brain pathologies. In vitro studies show that its loss compromises lysosomal function, contributing to cell death. To understand the role of ATP13A2 dysfunction in disease, we disrupted its expression through a viral vector-based approach in nonhuman primates. Here, in this pilot study, we injected bilaterally into the substantia nigra of macaques, a lentiviral vector expressing an ATP13A2 small hairpin RNA. Animals were terminated five months later, and brains were harvested and compared with historical non-injected control brains to evaluate cerebral pathological markers known to be affected in KRS and PD. We characterised the pattern of dopaminergic loss in the striatum and the substantia nigra, the regional distribution of α-synuclein immunoreactivity in several brain structures, and its pathological status (i.e., S129 phosphorylation), the accumulation of heavy metals in nigral sections and occurrence of lysosomal dysfunction. This proof-of-concept experiment highlights the potential value of lentivirus-mediated ATP13A2 silencing to induce significant and ongoing degeneration in the nigrostriatal pathway, α-synuclein pathology, and iron accumulation in nonhuman primates.

## Introduction

The *ATP13A2* gene (OMIM#610513) encodes a transmembrane lysosomal P5-type ATPase. The first mutations described in *ATP13A2* cause an autosomal recessive form of early-onset parkinsonism called Kufor-Rakeb Syndrome (KRS, OMIM#606693)^1^ and neuronal ceroid lipofuscinosis (NCL, OMIM#256730), later assigned to PARK9 locus. The clinical spectrum of KRS comprises parkinsonism, dystonia, supranuclear gaze palsy, and severe cognitive decline^2^. Brain MRI of KRS patients revealed generalised atrophy and putaminal and caudate iron accumulation, classifying KRS amongst neurodegeneration with brain iron accumulation (NBIA)^3^. NCL is a lysosomal storage disorder (LSD) that belongs to a genetically heterogeneous group of rare neurodegenerative diseases resulting from an accumulation of lipopigments, predominantly in the brain and retina. Typical clinical manifestations of these LSDs include progressive motor and cognitive impairment, seizures, and visual impairment or blindness. The outcome is invariably fatal, usually during the second or third decade.

Today, there is a growing list of homozygous and compound-heterozygous mutations^4,5^ linked to the truncation of the ATP13A2 protein, resulting in a loss of its functional capabilities. Remarkably, all mutations associated with KRS that have undergone experimental scrutiny exhibit varying degrees of interference with ATP13A2 function. These disruptions are brought about through diverse mechanisms, including nonsense-mediated messenger RNA decay, protein mislocalization, and premature degradation of protein products by the proteasomal system. However, the phenotypic spectrum associated with genetic variants in *ATP13A2* now becomes more and more complex since the (partial) loss of function leads to a whole range of clinical phenotypes, including previously comprised of KRS, NCL, multiple system atrophy (MSA)^6^, hereditary spastic paraplegia (SPG78)^7–10^, and juvenile-onset amyotrophic lateral sclerosis (ALS)^11^, as supported by both genetic and functional data, which makes the *ATP13A2* gene a puzzling gene^12^. To date, neuroprotective strategies for all those diseases that would stop or slow down the yet unrelenting ATP13A2-related degenerative process are eagerly awaited.

Mechanistic studies have previously reported that KRS-linked mutations in *ATP13A2* lead to several lysosomal alterations in *ATP13A2* KRS patient-derived fibroblasts, including impaired lysosomal acidification, decreased proteolytic processing of lysosomal enzymes, reduced degradation of lysosomal substrates and diminished lysosomal-mediated clearance of autophagosomes^13–16^. In addition, ATP13A2 levels are decreased in nigral dopaminergic neurons from sporadic Parkinson’s disease (PD) patients^13^, a result later corroborated by others^17^. Overall, those results unravel an instrumental role for ATP13A2 on lysosomal function and cell viability. Recently, the cellular function of this P5-type ATPase was unmasked^16^ as a lysosomal H^+^, K^+^-ATPase, and polyamine exporter, confirming its link to protein degradation via the lysosomal machinery^13–15,18^ and metal homeostasis (as reviewed in^19^), which cryo-electron microscopy has further structurally characterised^20–24^, altogether improving our understanding behind ATP13A2-associated functions.

To date, only one human brain neuropathological study from one ATP13A2-mutant KRS patient is available^2^, reporting widespread neuronal and glial lipofuscin accumulation, no Lewy-body pathology and α-synuclein pathology and sparse iron deposits in several brain areas, with skin and muscle containing electron-dense lamellated structures in this patient^25^. To decipher the pathological consequences of ATP13A2 deficiency, a vast spectrum of model organisms has been used, from yeast^26^ to different animal species (fly^27^, worm^28^, zebrafish^29^, and mouse^30,31^). Atp13a2 null mice exhibit increased autofluorescence in multiple brain regions, indicative of lipofuscin accumulation and modest age-related motor dysfunction preceded by neuropathological changes, including gliosis, accumulation of ubiquitinated protein aggregates, and endolysosomal abnormalities. Still, no overt neurodegeneration and iron accumulation have been observed. Additionally, a recessive mutation has been described in canine ATP13A2, causing adult-onset NCL in Tibetan terriers where autofluorescent cytoplasmic inclusions were detected in brain tissue consistent with NCL^32^. Altogether, model organisms used until now with the (partial) loss of ATP13A2 exhibit subtle age-dependent motor dysfunction, which at the cellular level correlates to lysosomal impairment. In mammalian model organisms, gliosis and lipofuscinosis were additional from ATP13A2 deficiency. These findings demonstrate the significance of lysosomal functionality at the core of ATP13A2 depletion. However, to truly decipher the clinical spectrum observed in humans, the role of ATP13A2 in neuronal survival, and how this dysfunction leads to neurodegeneration, we hypothesised that, in nonhuman primates, a species phylogenetically closer to humans, targeted silencing of ATP13A2 in dopaminergic neurons might result in neuronal death, α-synuclein pathology, metal dyshomeostasis, and autophagy dysfunction.

To this end, in a proof-of-concept pilot study, we injected bilaterally into the substantia nigra (SN) of macaques a lentiviral vector-mediated short-hairpin RNA (LV-shRNA) to downregulate the expression of ATP13A2 under the control of a ubiquitous promoter. Five months after administration, we observed nigrostriatal neurodegeneration associated with α-synuclein pathology, nigral iron accumulation, and autophagy lysosomal pathway (ALP) disturbances upon LV-mediated ATP13A2 silencing.

## Results

### Nigral ATP13A2 depletion induces nigrostriatal neurodegeneration

**Fig. 1:**
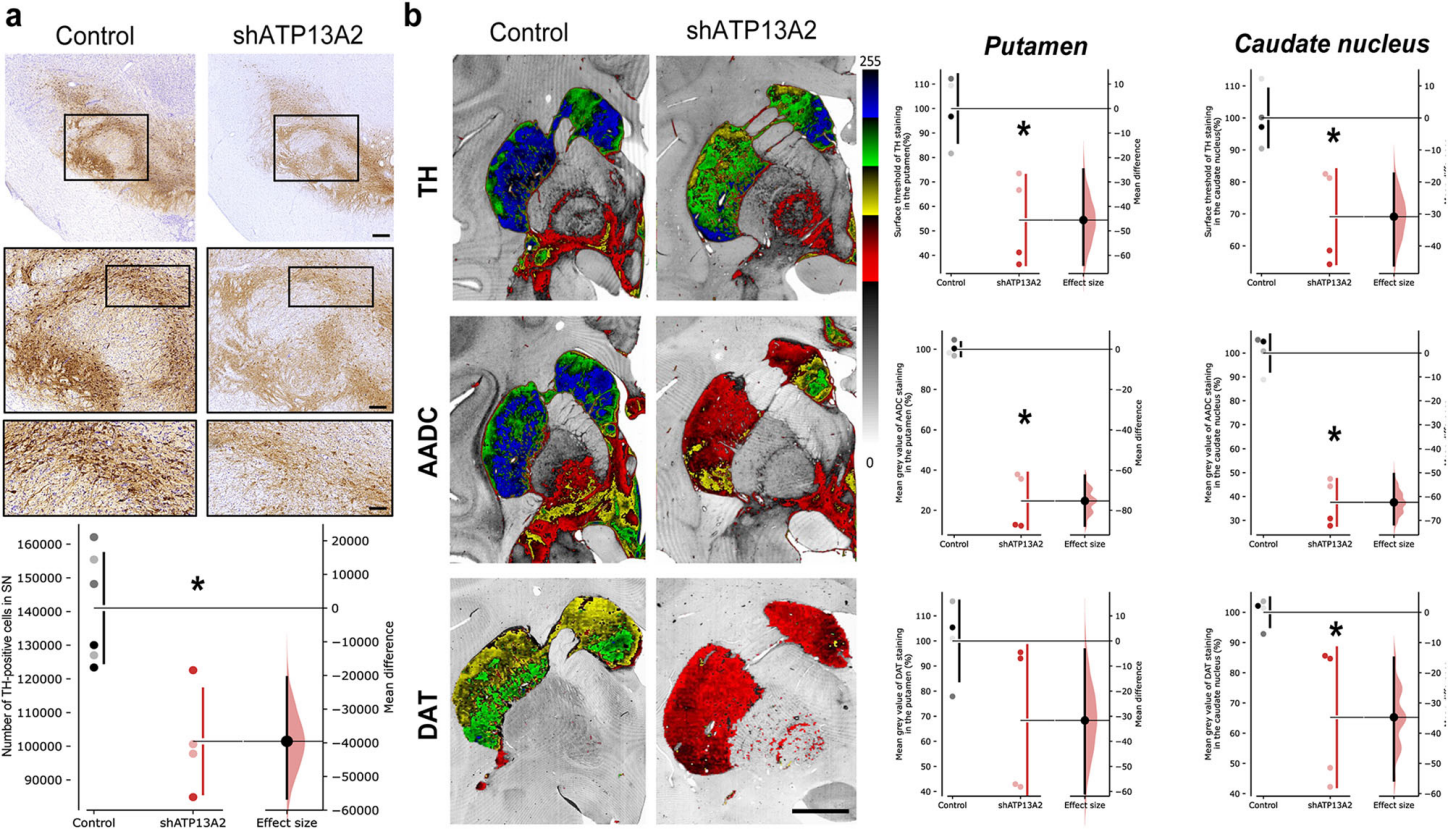
Decreased ATP13A2 levels in midbrain dopamine neurons lead to a PD-like pattern of nigrostriatal degeneration. **a** Representative images (top) and quantification (bottom) of Tyrosine Hydroxylase (TH)-positive neurons by stereological counting in the substantia nigra (SN) of control and shATP13A2-injected macaques five months after injection (SNpc: *p* = 0.0025, *t* = 3.814). Scale bar: 500 μm (top) 200 µm (inset). **b** Illustrative images (left) and scatter plots (right) of striatal TH, AADC and DAT immunostaining in control and shATP13A2-injected macaques, expressed as a percentage of controls (TH put: *p* = 0.0038, *t* = 3.947; TH cd: *p* = 0.00605, *t* = 3.548; AADC put: *p* = 0.0001, *t* = 10.45; AADC cd: *p* = 0.0001, *t* = 10.04; DAT put: *p* = 0.0557, *t* = 1.866; DAT cd: *p* = 0.0129, *t* = 2.944). A green fire blue LUT (lookup table) was used to enhance contrast and highlight the differences between non-injected and shAP13A2-injected macaques at the striatum level. Scale bars = 5 mm. Each dot represents one hemisphere of the control (black) and shATP13A2-injected NHPs (red) (light red is one macaque/deep red is the second macaque). The horizontal line indicates the average value per group ± SD. The bootstrapped mean difference with 95% CI (error bar) is shown on the right side of each graph. Comparisons were made using unpaired *t*-tests, *p < 0.05.

The lentiviral vector (LV) system is highly effective for delivering short hairpin RNA (shRNA) expression cassettes^33^ associated with no indications of increased non-human primate (NHP) tissue damage or inflammatory responses^34^. We used a functional and efficient sequence of shRNA targeting human ATP13A2 cloned into a LV plasmid, which displayed a 95% reduction in ATP13A2 levels by immunoblotting, as previously reported^13^. In addition, we showed that LV-mediated ATP13A2 knockdown in primary mesencephalic dopaminergic neurons resulted in neurotoxicity^13^. After confirming that lentiviral injection of non-targeting (Scramble) shRNA did not induce dopaminergic neurodegeneration in SN of macaques five months after injection (Supplementary Fig. 1), we decided to give priority to the LV encoding shATP13A2 to be injected in both hemispheres to reduce animal use. To further determine the pathological consequences of silencing ATP13A2 in a species closer to humans, adult female macaques received bilateral stereotactic injections (10 µl per hemisphere) of LV encoding shATP13A2 into the SN (Supplementary Fig. 2a). Tissues from 4 to 6 additional age- and sex-matched non-injected historical control animals were processed, stored and used under the same conditions for post-mortem experiments^35,36^. Five months after administration, macaques were tested to evaluate the integrity of the nigrostriatal pathway. ATP13A2 immunofluorescence revealed a decrease in ATP13A2 levels in Tyrosine Hydroxylase (TH)-positive dopaminergic neurons of the shATP13A2 versus the control group (Supplementary Fig. 2b). We observed a downward trend in protein levels, with an estimated reduction of around ~60% in endogenous ATP13A2 five months after shATP13A2 LV injection obtained by immunoblot of homogenised formalin-fixed tissues collected on sections from the SNpc (Supplementary Fig. 2c). Stereological counts showed that shATP13A2-injected animals exhibited a ~28% TH-positive cell loss in the SNpc (Fig. 1a) with a typical morphology of dying dopaminergic neurons displaying shrunken eosinophilic cytoplasm and cytoplasmic vacuolation, as signs of oxidative stress and autophagic degeneration^37,38^ (Supplementary Fig. 3). However, no overt parkinsonism was observed because the extent of the lesion remained below the threshold for the onset of motor symptoms, i.e., 45% cell loss^39^. At the striatal level, LV-shATP13A2 injection induced a significant 30% loss of TH immunoreactivity in the caudate nucleus (Fig. 1b) and 45% in the putamen (Fig. 1b). AADC, the enzyme that decarboxylates L-DOPA into dopamine (Fig. 1b), and striatal DAT immunostaining (Fig. 1b) were also both reduced, with a mean difference between groups of 60-70% (AADC) and 35% (DAT) in the caudate and putamen respectively.

### ATP13A2 depletion alters α-synuclein homeostasis in substantia nigra

**Fig. 2:**
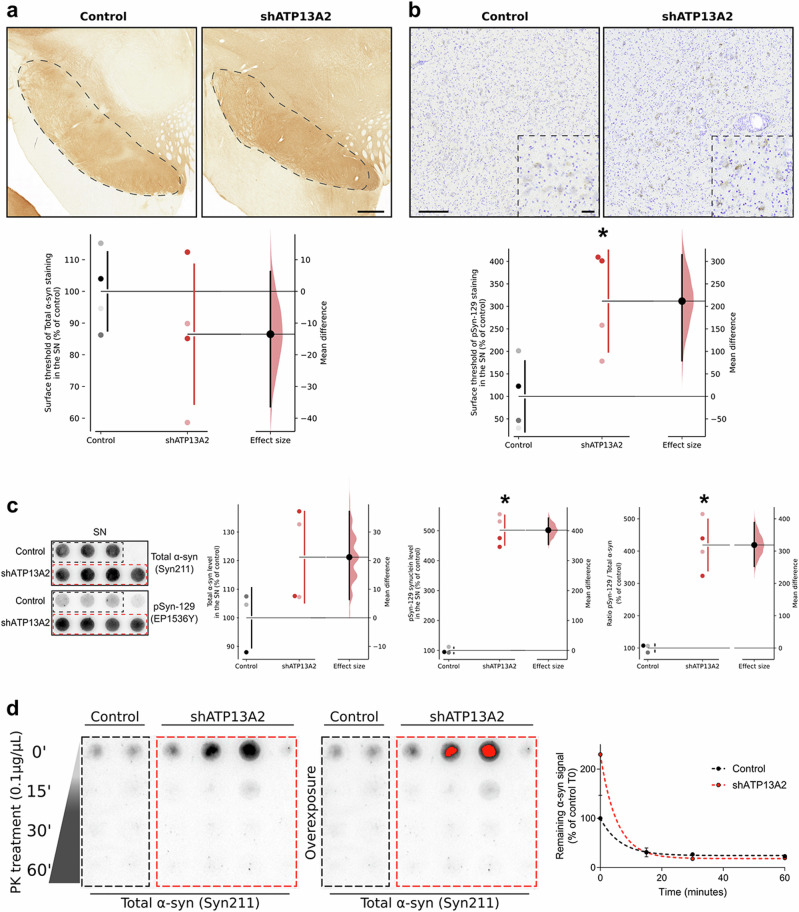
Decreased ATP13A2 levels induce α-syn pathology in substantia nigra. **a** Representative nigral sections of endogenous α-syn immunostaining (top) and scatter plot (bottom) of the mean grey value of syn211 immunostaining in the substantia nigra of control and shATP13A2-injected NHPs by quantification of surface staining, expressed as a percentage of controls macaques (*p* = 0.16355, *t* = 1.067). Scale bar: 200 µm. **b** Illustrative photomicrographs (top) and quantification (bottom) of phosphorylated S129 α-synuclein in the SNpc of control and shATP13A2-injected NHPs (*p* = 0.0109, *t* = 3.077). Scale bars = 100 µm (sections) and 10 µm (insets). **c** Midbrain protein extracts were analysed by dot-blot assay on nitrocellulose membrane and revealed by immunoblot with syn (*p* = 0.0528, *t* = 1.972) and pSyn (*p* = 0.0001, *t* = 13.39) antibodies. **d** Midbrain protein extracts were treated with proteinase K (PK) (0.1 µg/ml) for 0, 15, 30 and 60 min and analyzed by dot-blotting with syn211 antibody. Each dot represents one hemisphere of the control (black) and shATP13A2-injected NHPs (red) (light red is one macaque/deep red is the second macaque). Bootstrapped mean difference with 95% CI (error bar) is shown on the right side of each graph. Comparisons were made using an unpaired *t*-test. *p < 0.05 compared to control animals.

We then investigated the consequences of ATP13A2 depletion-induced nigrostriatal degeneration on nigral α-synuclein (α-syn) protein expression. We performed a quantitative post-mortem analysis of total α-syn (syn211 clone antibody) and phosphorylated α-syn at S129 (EP1536Y clone antibody) levels as a surrogate marker of the presence of α-syn pathology^40,41^ within the SN. In contrast to predictions from in vitro studies, we observed no changes in total α-syn-positive immunostaining in the SN (Fig. 2a*)*, while the α-syn pathogenic form, i.e., S129 phosphorylated α-syn, increased in DA neurons in shATP13A2-injected animals (Fig. 2b). Even though we could not obtain fresh tissues from the substantia nigra of macaques for Western blotting analysis, we performed protein extraction from formalin-fixed substantia nigra to further investigate α-syn pathology. Notably, our findings indicated that there were no discernible changes in total α-syn levels when using the syn211 antibody on mesencephalic total protein extracts, aligning with the results obtained from the immunohistochemical analysis, which also showed a significant accumulation of phosphorylated α-synuclein (pSyn) (Fig. 2c). The absence of modifications in total α-syn levels could potentially be attributed to (i) an increase in its phosphorylation state and/or (ii) a conformational alteration in the α-syn protein, causing it to adopt a pathological form that conceals the syn211 epitope (located within amino acids 121–125)^42^. Consequently, this post-translational modification might alter its conformation and render it undetectable by the syn211 antibody while remaining accessible to the anti-p-S129 α-synuclein antibody. This phenomenon could account for the observed increase in one species coinciding with no changes in the other. To get insight on the ratio of soluble versus aggregated α-syn, mesencephalic total protein extracts were subjected to Proteinase-K (PK) digestion and to dot-blotting with α-syn antibody (syn211 antibody) (Fig. 2d). No clear accumulation of α-syn PK-resistant aggregates was observed in the shATP13A2 group compared to control.

To further examine the distribution of α-syn, we performed triple immunofluorescence at the nigral level with the well-established dopaminergic marker TH, the lysosomal marker LAMP1 and the Syn211 antibody (Supplementary Fig. 4). We observed clustered compartments stained for LAMP1 with some clustering with α-syn in dopaminergic neurons of shATP13A2-injected animals compared to controls. Altogether, these results indicate no discernible changes in total α-syn or PK-resistant α-syn levels but increased pSyn protein expression in SN five months after nigral LV-shATP13A2 injection.

### Accumulation of lysosomal-related vesicles in dopaminergic neurons of shATP13A2-injected animals

**Fig. 3:**
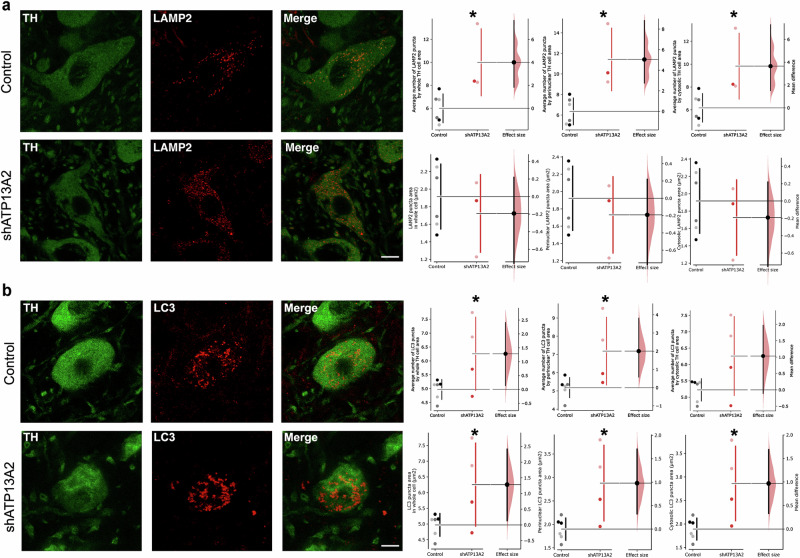
Accumulation of lysosomal-related structures after reduction of ATP13A2 levels. **a** Representative nigral sections of endogenous LAMP2 immunostaining in TH-positive cells (left) and scatter plot (right) depicting the average number of LAMP2-positive puncta in the whole cell area (*p* = 0.0099, *t* = 3.004), the perinuclear (*p* = 0.0042, *t* = 3.631) and cytosolic (*p* = 0.0139, *t* = 2.768) area as well as the LAMP2-positive puncta area in the whole cell (*p* = 0.2547, *t* = 0.6951), the perinuclear (*p* = 0.0256, *t* = 0.6907) and cytosolic (*p* = 0.25605, *t* = 0.6905) area. **b** Representative nigral sections of endogenous LC3 immunostaining in TH-positive cells (left) and scatter plot (right) depicting the average number of LC3-positive puncta in the whole cell area (*p* = 0.02435, *t* = 2.322), the perinuclear (*p* = 0.0172, *t* = 2.546) and cytosolic (*p* = 0.0382, *t* = 2.034) area as well as the LC3-positive puncta area in the whole cell (*p* = 0.01025, *t* = 2.880), the perinuclear (*p* = 0.01025, *t* = 2.882) and cytosolic (*p* = 0.00995, *t* = 2.900) area. Scale bar: 10 µm. Each dot represents one hemisphere of the control (black) and shATP13A2-injected NHPs (red) (light red is one macaque/deep red is the second macaque). Bootstrapped mean difference with 95% CI (error bar) is shown on the right side of each graph. Comparisons were made using an unpaired *t*-test. *p < 0.05 compared to control animals.

In vitro studies suggest that loss of ATP13A2 function leads to a rise in the number and size of lysosomes^13,14^, potentially as compensation for poor lysosomal function. Thus, we evaluated lysosomal-related vesicle number, surface and intracellular distribution in surviving nigral TH-positive neurons in vivo by segmentation analysis of confocal images. Following cell segmentation into whole cell area, perinuclear and cytosolic regions of interest (ROI) and detection of LAMP2- and LC3-positive vesicles, we calculated the average number and area of LAMP2- and LC3-positive puncta (Fig. 3). Consistent with in vitro reports, we found an increased number of LAMP2-positive lysosomes (Fig. 3a) and an increased number and surface of LC3-positive autophagosomes (Fig. 3b) in surviving nigral TH-positive neurons, consistent with a perturbation of these organelles. Strikingly, we observed an accumulation of both LAMP2- and LC3-positive puncta in the perinuclear area, which might indicate an accretion of immature and undegraded autolysosomes at this location as ATP13A2 inactivation is associated with impairment of autophagosome-lysosome fusion^27^. These findings are consistent with previous in vitro studies and may reflect compensatory changes in the ALP following improper clearance of lysosomal substrates. Lipofuscin is a pigmented, heterogeneous byproduct of failed intracellular catabolism conventionally found within lysosomes or the cytosol in the brains of patients with *ATP13A2* mutations^25^. We next attempted to examine the presence of lipofuscin in the nigral sections. Compared with control animals, LV-shATP13A2 macaques showed an increased deposition of autofluorescent storage material in the substantia nigra five months after nigral LV-shATP13A2 injection (Supplementary Fig. 5). Overall, these data indicate a dysfunction of the ALP associated with an accumulation of lipofuscin deposits characteristic of NCL.

### ATP13A2 depletion alters metal homeostasis in substantia nigra

**Fig. 4:**
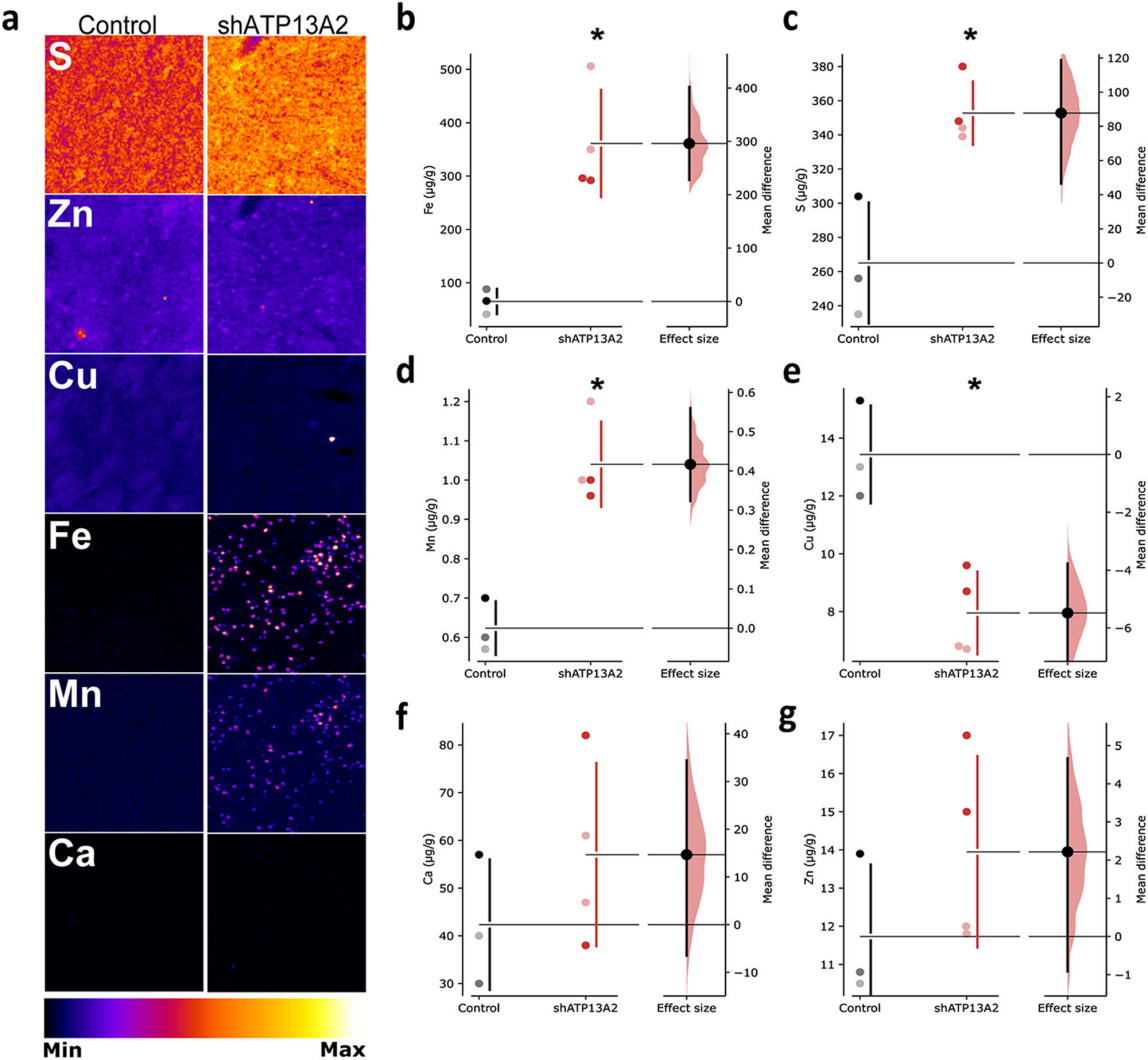
Decreased ATP13A2 levels alter SN heavy metal concentrations. **a** Representative elemental mapping in nigral slices of the macaque groups. Levels of iron (**b**), sulfur (**c**), manganese (**d**), copper (**e**), calcium (**f**), and zinc (**g**) were measured using synchrotron X-ray fluorescence in the SN of control and shATP13A2-injected macaques (Iron: *p* = 0.0022, *t* = 4.908; Zinc: *p* = 0.1292, *t* = 1.275; Copper: *p* = 0.0028, *t* = 4.644; Calcium: *p* = 0.15715, *t* = 1.118; Manganese: *p* = 0.0011, *t* = 5.785; Sulfur: *p* = 0.0038, *t* = 4.322). Each dot represents one hemisphere of the control (black) and shATP13A2-injected NHPs (red) (light red is one macaque/deep red is the second macaque). Bootstrapped mean difference with 95% CI (error bar) is shown on the right side of each graph. Comparisons were made using an unpaired Student’s *t*-test. *p < 0.05 compared to control animals.

Because the loss of ATP13A2 function is associated with dysregulated metal homeostasis in different experimental models^26,28,43,44^ and KRS patients^45,46^, we measured nigral heavy metal levels by synchrotron radiation x-ray fluorescence (SR-XRF) (Fig. 4a) as previously described^47^. Iron (Fig. 4b), sulfur (Fig. 4c), and manganese (Fig. 4d) concentrations were significantly increased in LV-shATP13A2 macaques compared to control animals. In the case of copper, concentrations were statistically decreased in LV-shATP13A2 macaques compared to control macaques (Fig. 4e), whereas no statistical differences were observed for calcium (Fig. 4f) and zinc (Fig. 4g). Interestingly, iron concentrations were a three-fold increase in shATP13A2 macaques, reminiscent of human pathology^45,46,48–50^. Next, we sought to determine the cell-type specificity and distribution of iron accumulation in the SN. We performed a triple immunofluorescence with the dopaminergic neuronal marker TH, the microglial marker Iba1, and Ferritin, the main iron-storage protein. We detected an increase of ferritin-positive cells in LV-shATP13A2 macaques compared to controls (Supplementary Fig. 6), and we observed increased expression in iron-storage protein ferritin in both Iba1-positive microglia (Supplementary Fig. 6a, d) and TH-positive neurons (Supplementary Fig. 6b, c). Upon closer examination, the iron-positive cells seemed to display distinctive morphological features consistent with activated microglia, including a compact soma and numerous thin processes^51^, indicative of an ongoing inflammatory response. This immunofluorescence analysis suggests that iron accumulates in dopaminergic neurons and microglial cells, with more abundant smaller puncta distributed in the microglia (Supplementary Fig. 6c, d).

## Discussion

**Fig. 5:**
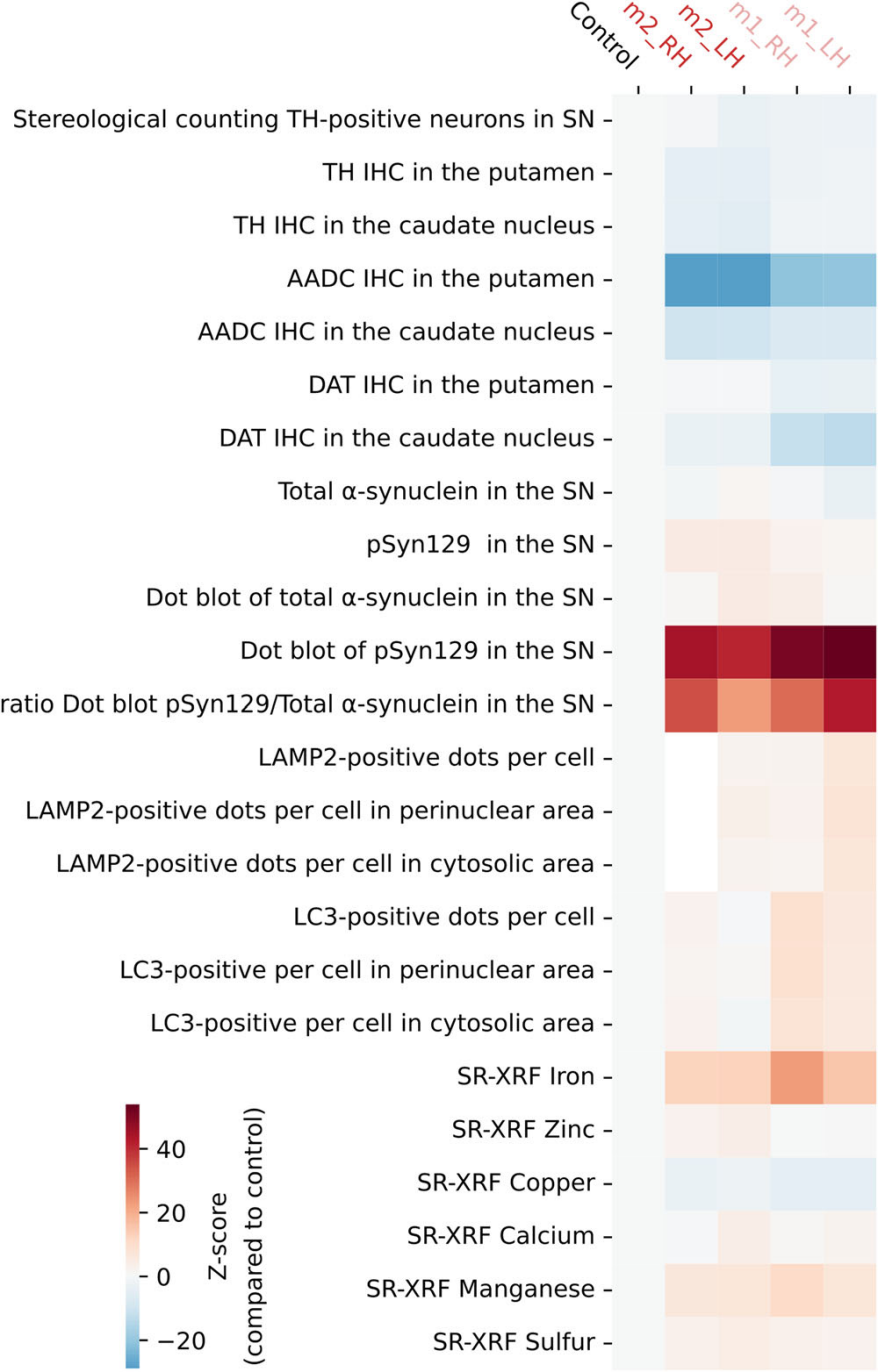
Direct comparison between experimental groups to highlight the pathological profile following the decrease ATP13A2 expression in the substantia nigra via a lentiviral shRNA approach. Heat map representing the normalized expression (Z scoring from control values) on the different variables used in this study, for each hemisphere of the two experimental NHPs. Controls are pulled as mean. From left to right: stereological counting Tyrosine Hydroxylase (TH)-positive neurons in the substantia nigra (SN) (stereo TH SN), TH, AADC and DAT immunostaining levels measured in the putamen and in the caudate nucleus (TH putamen; TH caudate; AADC putamen; AADC caudate; DAT putamen; DAT caudate), α-syn and pSyn signals in the SN (syn211 SN, pSyn SN, DB syn211 SN, DB pSyn SN, ratio pSyn/Syn211), the average number of LAMP2-positive puncta and LC3-positive puncta in the whole cell, perinuclear and cytosolic areas (LAMP2 nb whole cell, LAMP2 nb perinuclear, LAMP2 nb cytosolic, LC3 nb whole cell, LC3 nb perinuclear, LC3 nb cytosolic), the heavy metals content in SN (Iron, Zinc, Copper, Calcium, Manganese, Sulfur). The colour bars represent the z-score value of the ratio of each brain region.

Decreased ATP13A2 expression in the substantia nigra through an LV-shRNA strategy leads to major pathological features in nonhuman primates reminiscent of PD pathology. Knockdown of ATP13A2 by ~60% in nigral dopaminergic neurons affected their integrity, as evidenced here by (i) changes of α-synuclein immunoreactivity associated with an increase of one of its pathological forms (i.e., S129 phosphorylation); (ii) a dyshomeostasis in heavy metals and (iii) occurrence of ALP alterations. All these features were prominent in the SN of shATP13A2-injected NHP, resulting in a loss of striatal dopaminergic terminals and SNpc dopaminergic neurons. Relevant to KRS, we also show that silencing of ATP13A2 induces iron accumulation in the substantia nigra. These observations indicate an instrumental role for ATP13A2 deficiency in lysosomal function, α-synuclein and heavy metal homeostasis, and eventually cell viability. This new PD model provides a mechanistic framework that recapitulates some key pathological features of ATP13A2-related diseases (Fig. 5).

However, it is essential to acknowledge the limitations inherent in this study, foremost among them being the relatively small cohort sizes, typically consisting of only 2 to 3 animals per group. Nevertheless, this size is commonly recognised as a standard size for pilot studies in non-human primate (NHP) research, grounded in scientific considerations and ethical constraints^52^. Notably, our study’s statistical power is diminished, a common characteristic in NHP research, where a significant proportion often involves minimal animal numbers per group, typically ranging from 2 to 4 individuals, due to cost and difficulty accessing macaques. With two groups of two to three macaques, either LV-shATP13A2-injected or non-injected historical controls, it is challenging to obtain for each variable homogenous results that allow giving definitive answers. Some endpoints do not reach statistical significance because of the small sample size. Hence, the importance of the Gardner–Altman plots that focus upon the effect size of the difference, when any. Many studies conducted by our team and other colleagues working on NHPs have generally used these types of low group sizes^53–63^. Our team is, however, known to involve a much larger number of NHPs when moving to definitive studies^47,64–67^ that have always confirmed experiments with lower n.

To investigate the pathological consequences of ATP13A2 deficiency, a vast panel of models of ascending complexity, i.e., in human dopaminergic neuroblastoma-derived cells, mesencephalic primary cultures, mice, and dogs, have been developed. However, in ATP13A2 knockout mice, behavioural phenotype impairment and pathophysiological abnormalities are not detectable until old age (20- to 29-month-old), without overt neurodegeneration^30,31^, suggesting the involvement of strong compensatory mechanisms in transgenic mice. Dogs with ATP13A2 mutations show clinical signs of NCL similar to those of KRS patients, but without parkinsonism, associated with astrogliosis and autofluorescent storage material in nervous and cardiac tissues at an average age of 6 years, progressively worsening over time^32,68,69^. A striking result of the current study is the efficacy of the induction of dopaminergic neurodegeneration over five months, corresponding to a short duration for a study in nonhuman primates. We hypothesise that follow-up long-term studies are required to observe the occurrence of a full spectrum of behavioural deficits, including progressive motor and cognitive impairment, i.e., when the threshold of the nigrostriatal lesion for manifest PD will be reached^39^. Additionally, we should also target other brain regions to extend the disease modelling panel associated with ATP13A2 loss of function. A second aspect to be envisioned for future studies is to compare the effects obtained with an adeno-associated virus (AAV)-based strategy. Several advantages can be emphasised by moving to AAV due to its unique biological and biophysical properties. First, AAV is compatible with the viral capacity to carry recombinant DNA such as shRNA. Second, AAV has a diffusion advantage over LV due to its particle size, which is 25 nm in diameter, whereas LV particles are no less than 100 nm in diameter. Third, specific serotypes of AAV share the ability to cross the blood-brain barrier. They may be delivered through various administration routes, making them a powerful tool for genetic material delivery to the CNS^70–72^. Fourth, AAV has proved safe and effective in preclinical and clinical settings and has been the virus of choice for developing gene therapies for PD^73^.

While this proof-of-concept study is encouraging, pending questions await future investigation and should be conducted with a larger sample size and a parallel control group to increase statistical power. To date, it is evident that numerous neurological disorders have been linked to ATP13A2 with diverse and complex neurological deficits, complicating our understanding of ATP13A2. However, the fact that mutations in a single lysosomal gene result in such a diversity of disease phenotypes suggests that lysosomal burden is a common thread running amongst them. Therefore, deciphering the ATP13A2-dependent cellular roles and pathways would enhance our understanding of brain physiology and the broader mechanisms underpinning neurodegeneration. First, one aspect to investigate in future studies will be the analysis of polyamine levels. Recent studies demonstrated the polyamine transport function of ATP13A2^16^, which may support heavy metal chelation^74^ and provide antioxidative effects^75^. Polyamine levels decline with ageing and are altered in PD patients. Analysing polyamine levels in this new experimental animal model should provide valuable information regarding the precise role of polyamines in PD, KRS, and beyond. Second, in humans, *ATP13A2* mutations cause neurodegeneration with brain iron accumulation^45^; in particular, KRS patients present iron deposits in the brain^45,46^ whereas, in mammalian cells, ATP13A2 expression and transport activity protect against several heavy metals, such as manganese, zinc, and iron toxicity^43,76^. Our study measured an iron increase of over 300%, demonstrating a close relationship between iron accumulation and ATP13A2 depletion. Several experiments proposed that the ferric iron load accelerates α-synuclein aggregation^77^, and an increase in iron levels plus a decrease in ferritin can contribute to nigral degeneration^78^. An increased iron burden has been found in early PD stages in the SN^48–50,78^. The pathological consequences of the increased iron SN burden could contribute to α-syn pathology, as we observed an increased nigral pSyn expression.

Noteworthy, we also observed a significant increase in manganese in the SN, consistent with the fact that ATP13A2 has been reported to be involved in manganese homeostasis^26,28,44^. This result is reminiscent of previous reports showing that ATP13A2 protects cells against manganese toxicity by transporting manganese into lysosomes; however, this protection is lost in the case of mutations in this gene^43^. A recent study showed that ATP13A2 polymorphism could be a risk marker for the neurotoxic effects of manganese in humans^79^. Both of our findings are of interest regarding the work of Fleming and colleagues that show increased manganese and iron in ATP13A2 knockout mice administered with manganese chloride compared to treated wild-type mice but also increased α-synuclein aggregation and higher lipofuscin deposits in the SN^80^. Of note, the copper reduction is also of interest and deserves further studies as it has been reported to be reduced in human SN dopaminergic neurons of PD and incidental Lewy body disease cases^81^.

Overall, we show that ATP13A2 depletion triggers PD/KRS-related features in nonhuman primates, with stimulating perspectives for understanding human ATP13A2 pathology per se. Further studies are needed to extend our observations and appreciate the full spectrum of behavioural and pathological outcomes of ATP13A2 deficiency. Understanding the complex contribution of the dysfunctional or absent lysosomal ATP13A2 protein to ALP dysfunction, heavy metal dyshomeostasis, and neurodegeneration would likely open new therapeutic opportunities for slowing down the degenerative process in PD/KRS patients.

## Methods

### Ethics statement

All experimental protocols comply with the Council Directive of 2010 (2010/63/EU) of the European Community and the National Institute of Health Guide for the Care and Use of Laboratory Animals. The proposed research has received approbation from the French Ethical Committee for Animal Research CE50 (agreement number: 50120102-s).

### Recombinant LV-shRNA plasmid and virus production

shRNA lentiviral plasmids were purchased from Sigma-Aldrich. shRNAs were validated based on the Genbank human ATP13A2 sequence NM_022089. All sequences were BLAST confirmed for specificity. Synthetic DNA encoding shRNA sequences targeting human ATP13A2 were cloned into shRNA lentiviral plasmids (pLKO.1-puro) under the control of the U6 promoter, which is a type III RNA polymerase III promoter commonly used for driving small hairpin RNA (shRNA) expression in vector-based RNAi. shRNA lentiviral plasmids were transfected into BE(2)-M17 cells for 48 h to determine the level of knockdown of gene expression by immunoblotting^13^. The ATP13A2 shRNA-expressing viruses have been tested as described above. The virus titers were identical and corresponds to 6.73 × 10^8^ pI/ml for ATP13A2 shRNA and Scramble shRNA sequence. The ATP13A2 shRNA sequence used in the study is ccggGCCCATCAACTTCAAGTTCTACTCGAGTAGAACTTGAAGTTGATGGGCtttttg and the Scramble shRNA sequence used in the study is ccggCAACAAGATGAAGAGCACCAACTCGAGTTGGTGCTCTTCATCTTGTTGttttt. Lentiviral vector production was done by the service platform for lentiviral vector production “Vect’UB’ of the TMB-Core of Bordeaux University. A lentiviral vector was produced by transient transfection of 293 T cells according to standard protocols. In brief, subconfluent 293 T cells were co-transfected with the lentiviral genome (psPAX2)^82^, with an envelope coding plasmid (pMD2G-VSVG) and with vector constructs by calcium phosphate precipitation. LVs were harvested 48 h post-transfection and concentrated by ultracentrifugation. Viral titers of pLV lentivectors were determined by transducing 293 T cells with serial dilutions of viral supernatant, and a control pLV-EGFP expression was quantified 5 days later by flow cytometry analysis or provirus copies number by qPCR method.

### Animals and stereotactic injections

Two female adult rhesus macaques (*Macaca mulatta*) weighing ∼6–7 kg (9–10 years old) were used in this study. The macaques were housed in the animal facility of the Institute of Neurodegenerative Diseases (UMR CNRS 5293) under standard conditions (12/12 h day/night cycle, ∼60% humidity, and ∼22 °C). Tissues from 4 to 6 additional age- and sex-matched historical control animals were used for post-mortem experiments when needed and available^35,36^. Bilateral intranigral LV injections of 10 µl were performed with electrophysiological guidance considering the line passing through the anterior (AC)-posterior (PC) commissures as a reference: -7mm posterior to AC, -4 mm below the AC-PC line, and 3 mm lateral from midline. At the end of the experiment (5 months post-injection), as previously described^47,62,66^, all macaques were euthanised by sodium pentobarbital overdose (150 mg/kg iv), followed by perfusion with saline solution (containing 1% heparin) and 4% paraformaldehyde, and brains were quickly removed and processed for histological studies. Brains were sectioned in 50 µm thick serial free-floating coronal sections in a Leica CM3050S cryostat (Leica Microsystems, Wetzlar, Germany) at −20 °C, collected in PBS Azide 0.2% and stored at 4 °C until they were processed for analysis.

### Histological analysis

#### Dopaminergic neurodegeneration assessments

To assess the effect of ATP13A2 silencing on dopaminergic neurones and fibres, TH (tyrosine hydroxylase), AADC (Aromatic L-amino acid decarboxylase), and DAT (Dopamine Transporter) immunohistochemistry and quantifications were performed on striatal and SN sections as previously described^47,62,66^. Briefly, 50-µm free-floating sections from one representative level of the striatum (post-anterior commissure) and serial sections (1 every 12) corresponding to the whole SNpc were selected. For TH staining, anterior striatum (post-anterior commissure) sections and a whole rostrocaudal series (1 to 12) encompassing the SNpc were selected. For AADC and DAT staining, anterior striatum (post-anterior commissure) sections were used. Sections were incubated with mouse monoclonal TH antibody (Millipore, MAB318, 1:5000), rabbit polyclonal AADC antibody (Merck AB136, 1:1000), or rat monoclonal DAT antibody (Merck MAB369, 1:500) for one night at room temperature (RT) and revealed the next day with the corresponding peroxidase EnVision secondary antibody, followed by DAB visualisation. SNpc sections were mounted on gelatine-coated slides, counterstained with 0.1% cresyl violet solution, dehydrated and cover-slipped. Striatal sections were mounted on gelatine-coated slides, dehydrated and cover-slipped. Striatal sections were analysed by optical density (OD) in the caudate nucleus and putamen. The slides were scanned using Epson Expression 10000XL high-resolution scanner. Images were analysed using ImageJ open-source software (version 1.53) to compare mean grey levels in the caudate nucleus and putamen. TH-positive neurons of the SNpc were counted by stereology blind about the experimental condition using a Leica DM6000B microscope coupled with the Mercator software (IMASCOPE, France). The SNpc was delineated for each slide, and dissector probes for stereological counting (100 × 80 µm spaced by 600 × 400 µm) were applied to the map obtained. Each TH-positive cell with the nucleus included in the probe was counted following the stereological rules of exclusion^47,62,66^. The total number of TH-positive neurons in the whole SNpc was then assessed per hemisphere using the optical fractionator method.

#### α-Synuclein pathology assessment

α-synuclein status was assessed with a mouse monoclonal antibody raised against human α-syn (ThermoFisher [Syn211], 32-8100, 1:1000) and another against phosphorylated α-syn (Abcam, [EP1536Y], ab51253, 1:5000) as previously described^47,62,66^. Briefly, selected sections of all animals were explicitly identified and incubated in the same well to allow direct comparison of immunostaining intensity. Sections were incubated overnight at room temperature with the antibodies. The following day, the revelation was performed with the corresponding peroxidase EnVision secondary antibody followed by 3,3′-diaminobenzidine (DAB, DAKO, K346811-2) incubation. Sections were then mounted on gelatinized slides, counterstained with 0.1% cresyl violet solution, if necessary, dehydrated, and cover-slipped until further analysis. Slides were scanned with a high-resolution scanner (Panoramic Scan II, 3DHISTECH Ltd, France) at x20 magnification and on five layers spaced by 1.4 µm each. Quantifications were estimated by immunostaining-positive surface quantification at regional levels with the Mercator software (IMASCOPE, France), as previously described^47,62,66^.

The “surface” is an additional quantification method used in tissue sections. For these analyses, a specific staining process was used to keep all tissues together in the same solution during the staining process. Then, high-resolution whole colour slide images were acquired with the 3D Histech Panoramic Scanner at 20X magnification, with 5 layers in extended mode. Each image was opened in the off-line MERCATOR PRO 7.12.3 software (Explora Nova, France), and all regions of interest were mapped. The same brightness and contrast rules were systemically applied to the RGB pictures to optimise the staining’s details without any image saturation. The colour thresholding tool was then used to select the threshold corresponding to the brown colour revealed by the DAB staining. The threshold was established based on the intensity of the staining to detect the maximum DAB staining. The file of the threshold parameters was saved and applied to all measurements for each animal/staining. Before performing the quantification, the threshold was randomly applied to some images of different treatment groups to verify the accuracy of the settings. In each region, the software extracted the surface corresponding to the threshold defined. The “surface” parameter was finally expressed as a ratio of the total surface of each area of interest. This parameter has been previously used and published^83^.

#### Immunofluorescence and image analysis

To assess ATP13A2 expression and autophagy-related markers (i.e., autophagosomes and lysosomes), nigral macaque sections were incubated simultaneously with two antibodies: a mouse monoclonal antibody against TH (Millipore, MAB318, 1:5000), and, either a rabbit monoclonal antibody against ATP13A2 (Novus Biological, NB110-41486, 1:250) or a rabbit monoclonal against human LAMP1 (Genetex, GTX62434, 1:1000) or LAMP2 (Santa Cruz Biotechnology, sc-18822, 1:1000) or LC3 (Novus Biological, NB100-2220, 1:1000) or human α-syn (ThermoFisher [Syn211], 32-8100, 1:1000) for one night at room temperature. Incubation with secondary antibodies was done sequentially with a goat anti-mouse AlexaFluor 488 (Invitrogen, A28751, 1:400) or a goat anti-rabbit AlexaFluor 568 (Invitrogen, A11036, 1:400) 1h30 at room temperature. Sections were mounted on non-gelatinised slides, and cover slipped using fluorescent mounting media without DAPI (Vector Labs). Images were acquired using a Zeiss SP5 confocal microscope.

For LAMP2 and LC3 count and area analysis in TH-positive cells at 5 months post-injection, image analysis was performed in Fiji/ImageJ. All image acquisitions and analyses were performed blinded to the researcher. Image analysis was performed in Fiji/ImageJ using custom scripts (available at https://github.com/Soria FN/Lysosome_analysis). Cells were segmented by manual cell selection using the TH channel. The mask was then applied to the LAMP2 and LC3 channels, where LAMP2 and LC3 staining was quantified by automatic thresholding followed by binarisation. Total staining was then normalised per cell. For subcellular localisation analysis, cytoplasmic and nuclear ROIs were manually segmented using the TH channel of single z-planes where the nucleus was clearly visible. To prevent a bias due to low cytoplasmic area and to ensure that the comparisons were made between cells with similar cytoplasmic ROIs, the analysis was subsequently performed only in cells where the ratio of cytoplasmic to nuclear ROI area was 2:1. Lysosomal-related puncta were segmented from the LAMP2 or LC3 channel, and the average number and area from the nucleus centre to the centre of mass of each lysosomal ROI was calculated. Finally, to estimate the distance to the nucleus border, the average radius of an ellipse fitted to the nuclear ROI was subtracted from the distance to the nucleus centre.

To assess ferritin pattern expression, each nigral macaque section punches was made from free-floating sections and mounted on the same slide to compare the pattern between TH, Iba1 and ferritin-expressing cells in SNpc. The resulting glass slide was incubated simultaneously with a mix of antibodies, including chicken anti-TH (Abcam, ref ab76442), goat anti-Iba1 (Abcam, ref ab5076) and rabbit anti-ferritin [EPR3004Y] (Abcam, ref ab75973), all diluted at 1:1000 and incubated for one night at room temperature. Incubation with secondary antibodies was done after thorough rinses, with a mix of donkey fluorescent secondary antibodies raised against the different species (respectively anti-chicken AlexaFluor 568 (Invitrogen, A11041), anti-goat AlexaFluor 647 (Invitrogen, A21447) and anti-rabbit AlexaFluor488 (Invitrogen, A21206) diluted at 1:500 and incubated 1 h at room temperature. After several rinses with PBS, a nuclear stain was made with 1:5000 diluted Hoechst solution for 30 s, and sections were then coverslipped using ProlongGlass fluorescent mounting media (Thermo Fischer, ref P36984). Images were acquired using a 3D-HISTECH Pannoramic Scan II with 20X objective and extended focus mode (5 layers and 7 steps).

#### Lipofuscin imaging

For imaging of lipofuscin, untreated free-floating nigral slices were mounted with a Vectashield Antifade Mounting Medium to observe lipofuscin autofluorescence. Z-stack images were acquired using a Leica DM6 CFS TCS SP8 confocal microscope at x63 magnification, with an excitation of 500 nm and an emission of 600–650 nm. Analysis was performed through ImageJ Fiji.

### Biochemical analysis

#### Protein Extraction and immunoblot analysis

We used 2 mm^3^ formalin-fixed tissue per animal. Tissue patches were extracted using the Qproteome FFPE TissueKit (Qiagen)^84–86^ and then quantified using the Lowry method (Biorad RC DC Protein Assay Kit) following the manufacturer’s instructions. Based on total protein concentrations calculated from the assay, aliquots of tissue lysates corresponding to 20 μg were prepared for each structure in Laemmli buffer (Tris-HCl 25 mM pH 6.8, glycerol 7.5%, SDS 1%, DTT 250 mM and bromophenol blue 0.05%).

For immunoblot analysis, samples were then boiled at 100 °C for 5 min and loaded into a 12% SDS-PAGE gel, followed by electrophoretic transfer onto 0.2 µm nitrocellulose membranes (Bio-Rad, Hercules, CA, United States). Membranes were blocked in phosphate buffer saline (PBS) with 5% dry skimmed milk and probed with an antibody against ATP13A2 (Novus Bio, NB110-41486, 1:1000) overnight. Anti-β-actin (1:10 000, Sigma, A5441) was used to control equal loading. Appropriate secondary antibodies coupled to peroxidase were revealed using a Super Signal West Pico Chemiluminescent kit (Immobilon Western, Chemiluminescent HRP substrate, Millipore) and analysed with Image Lab software 5.0 (Bio-Rad, Hercules, United States).

#### Dot blotting analysis

The dot blot analysis was performed as previously described^87^. After heating at 100 °C for 5 min, 20 μg of protein extract was diluted in buffer (25 mM Tris-HCl, 200 mM Glycine, 1% SDS) and filtered through a nitrocellulose membrane (Bio-Rad, 0.2μm pore size). Membranes were then saturated in 5% dry milk before incubation with total α-syn (1:1000, ThermoFisher [Syn211], 32-8100) and anti-phosphorylated-α-syn at Ser129 (1:5000, Abcam [EP1536Y], ab51253). To evaluate proteinase K-resistant α -syn contained in midbrain protein extracts from control and shATP13A2-injected animals, we subjected 50 μg of protein extract to digestion with proteinase K (0.1 µg/ml) for 0, 15, 30, and 60 min. The reaction was stopped by boiling for 5 min at 100 °C before dot blotting with syn211 antibody. Revelation was done as described above.

### SR-XRF microscopy elemental mapping of brain tissue cryosections

The synchrotron experiments were carried out at the Diamond Light Source, Harwell Science and Innovation Campus (Didcot, UK) with 3-GeV energy of the storage ring and 300-mA currents with top-up injection mode. All SR-XRF microscopy investigations reported herein were carried out on the microfocus spectroscopy beamline (I18). The micro-XRF elemental mapping was acquired at room temperature with an incident x-ray energy set to 12 keV using a Si(111) monochromator. This resulted in an x-ray photon flux of 2 × 10^11^ photons/s. The SN cryostat section (50 µm in thickness) of each animal was collected from free-floating sections and mounted onto an x-ray transparent metal-free 4-µm-thick Ultralene foil (SPEX CertiPrep, Metuchen, NJ, USA) secured to a customised polyetheretherketone (PEEK) holder ensuring contamination-free samples and reduced x-ray scattering contribution. The samples were then left and kept at room temperature in a moisture-free box until used for analysis. The samples were affixed to a magnetic plate connected to the sample stage of the microfocus beamline. The four-element Si drift Vortex ME4 energy-dispersive detector (Hitachi High-Technologies Science America), with Xspress 3 processing electronics, was operated in the 90° geometry; hence, it minimizes the background signal. The sample-detector distance was fixed (75 mm). The sample was held at 45° to the incident X-ray beam and raster in front of the beam while the XRF spectra were collected. An area of 500 µm by 500 µm within the SNpc was mapped for each sample with a step size that matches the beam size (5 µm) and a dwell time of 1 s per pixel due to the low concentration of the element. A thin (100 µm) pellet of the National Institute of Standards and Technology (NIST) standard reference materials SRM1577c (bovine liver material, NIST, Gaithersburg, MD, USA) was measured to calibrate experimental parameters as well as a thin-film XRF reference material (AXO DRESDEN GmbH) with nanoscale uniform mass depositions in the range of ng/mm^2^. This was followed by elemental quantification calculated using the Fundamental Parameters (FP) approach through the open-source software PyMCA^88^, in which the reference material and the sample are modelled in terms of main composition, density, and thickness. The fluorescence spectrum obtained from each pixel was fitted, the elemental concentration (micrograms per gram of dry weight or parts per million) maps were generated, and an average elemental concentration of the SNpc regions was obtained.

### Statistical analysis

For all experiments, comparisons among means were performed using raw data Student’s two-tailed unpaired *t*-test (GraphPad Prism 10.0, San Diego, CA). Statistical significance was set at *p* < 0.05. The debate about the need to move beyond *p*-value is raging. Data must be further analysed with estimation graphics^89^ emphasising the effect size. Therefore, all data appear as estimation graphics called ‘Gardner–Altman plots’: on the left of each graph, data of controls and sh-ATP13A2 groups are presented as scatter plots showing the observed values along with the defined descriptive statistics (mean ± standard deviation). Each dot represents one hemisphere of the control (shade of black) and shATP13A2-injected NHPs (shade of red). One circle corresponds to one hemisphere. The dark circles correspond to the control group, and the red circles correspond to the shATP13A2-injected groups (Light red is one macaque/deep red is the second macaque). On the right of each graph is a contrast graph using the different axes to display an effect size, which means difference. Horizontally aligned with the mean of the test group, the mean difference is indicated by the black circle. The black vertical line illustrates the 95% confidence interval (CI) of the mean difference. Given the observed data, the curve shows the resampled distribution of the effect size.

### Supplementary information

Supplementary Information

Supplementary Table 1

## Data Availability

The data supporting the findings of this study are provided in Supplementary Table 1. The material supporting the findings of this study is available from the corresponding author on request.
