# Supplementary material for: Nigral ATP13A2 depletion induces Parkinson’s disease-related neurodegeneration in a pilot study in non-human primates
Source: NPJ Parkinsons Dis. 2024 Aug 1;10:141. doi: 10.1038/s41531-024-00757-4 (PMC11294619; doi:10.1038/s41531-024-00757-4)
Supplement: Supplementary file 1 — Supplementary Information [file 41531_2024_757_MOESM1_ESM.docx]

**Supplementary Figures**

**
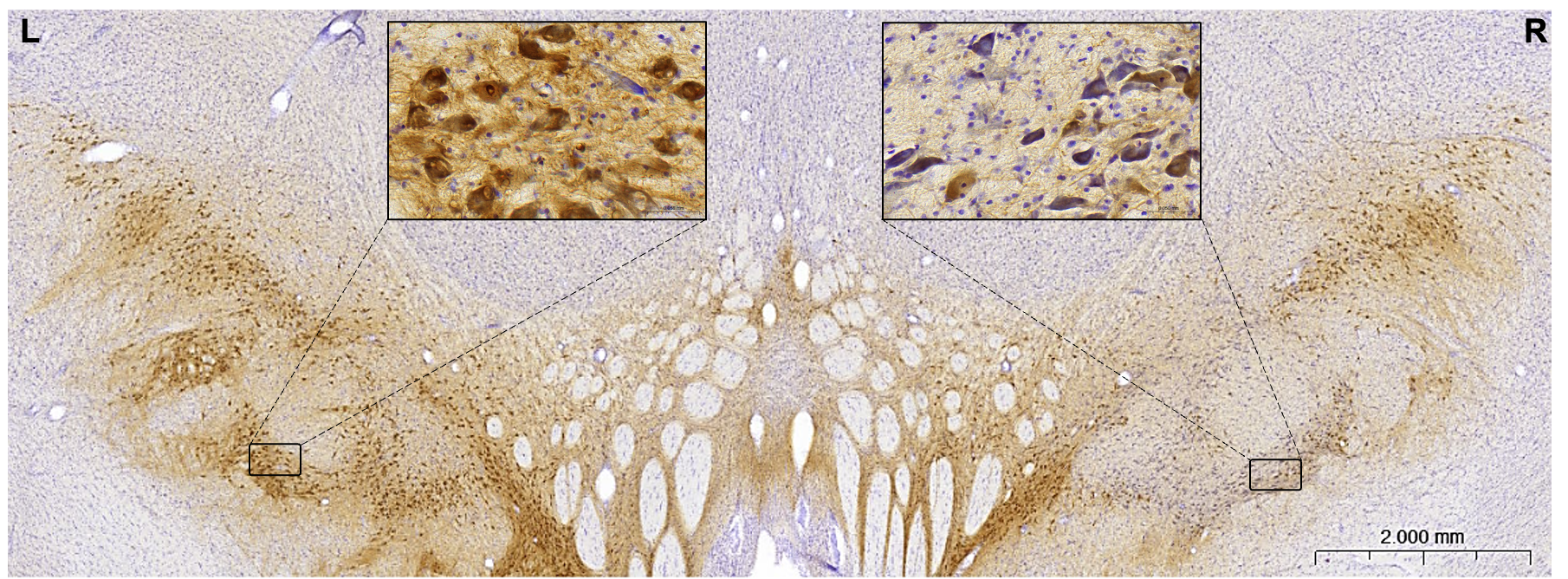
**

**Supp. Figure 1. Intranigral lentiviral delivery of a shScramble lentiviral vector does not induce dopaminergic neurodegeneration in a macaque.** **(a)** Representative photomicrographs of TH-immunostained (brown) thionin-counterstained (blue) SN from shSramble (shRNA-targeting scramble)-injected hemisphere (left; L) and shATP13A2-injected hemisphere (right; R) five months after injection in macaques. Stereological counts showed that shATP13A2-injected hemisphere exhibited TH-positive cell loss in the SNpc (35%, shATP13A2-injected hemisphere, 108,256 TH-positive neurons (right; R); shScramble-injected hemisphere, 165,107 TH-positive neurons (left; L)). Scale bar = 2 cm.

**
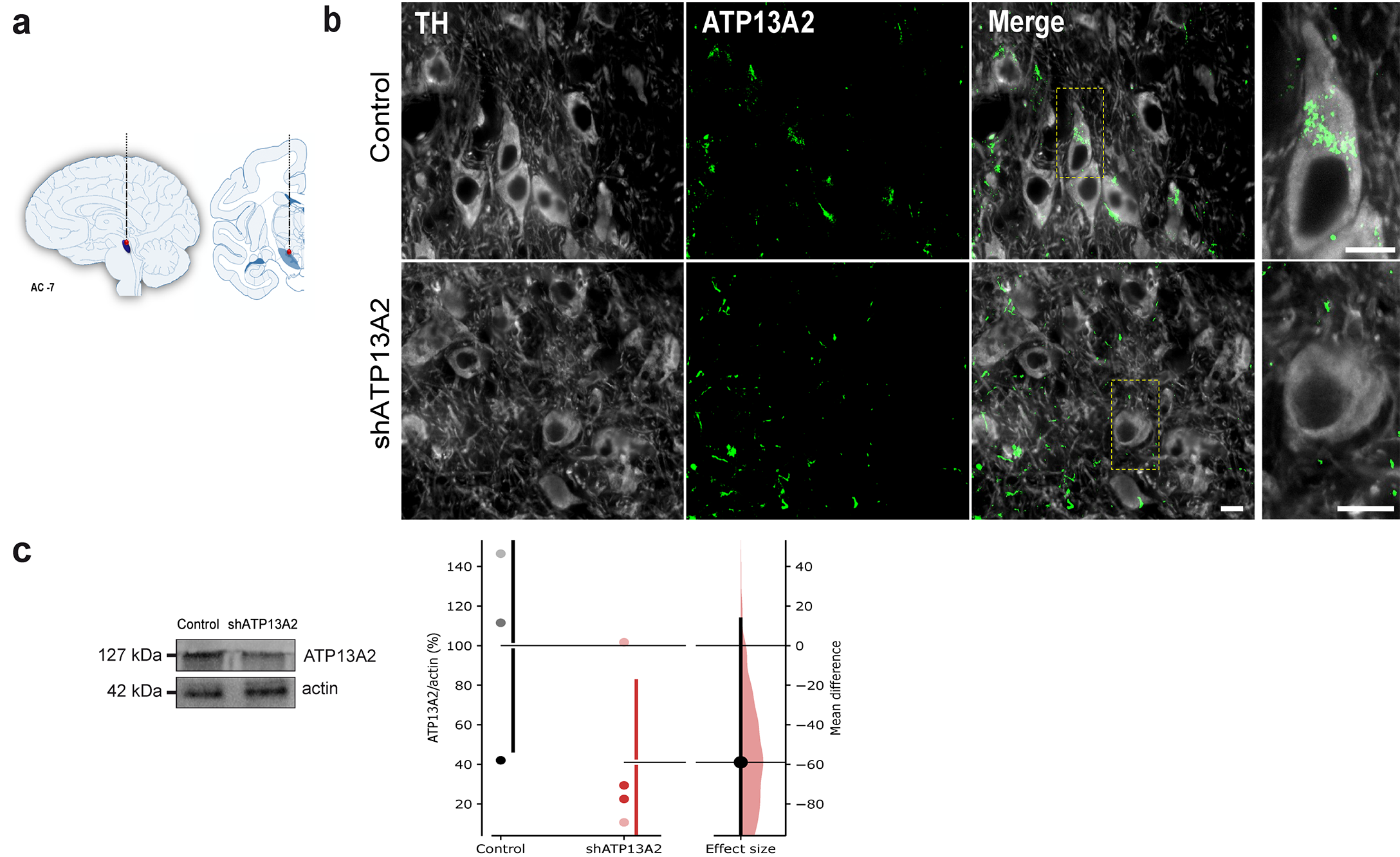
**

**Supp. Figure 2. Intranigral injection of a shATP13A2 lentiviral vector decreases ATP13A2 expression levels.** **(a)** Schematic diagram indicating the site of nigral stereotactic shATP13A2 injection in macaques. **(b)** Representative confocal micrographs of double immunostaining for dopaminergic marker tyrosine hydroxylase (TH) and the ATP13A2 protein in the substantia nigra of control and shATP13A2-injected macaques five months post-administration. Scale bar = 10 μm. **(c)** Immunoblot representative image and analysis of ATP13A2 protein levels in the substantia nigra of control and shATP13A2-injected macaques (p=0.07825, t=1.666). Each dot represents one hemisphere of the control (black) and shATP13A2-injected NHPs (red). Bootstrapped mean difference with 95% CI (error bar) is shown on the right side of each graph. Comparisons were made using an unpaired Student's t-test.


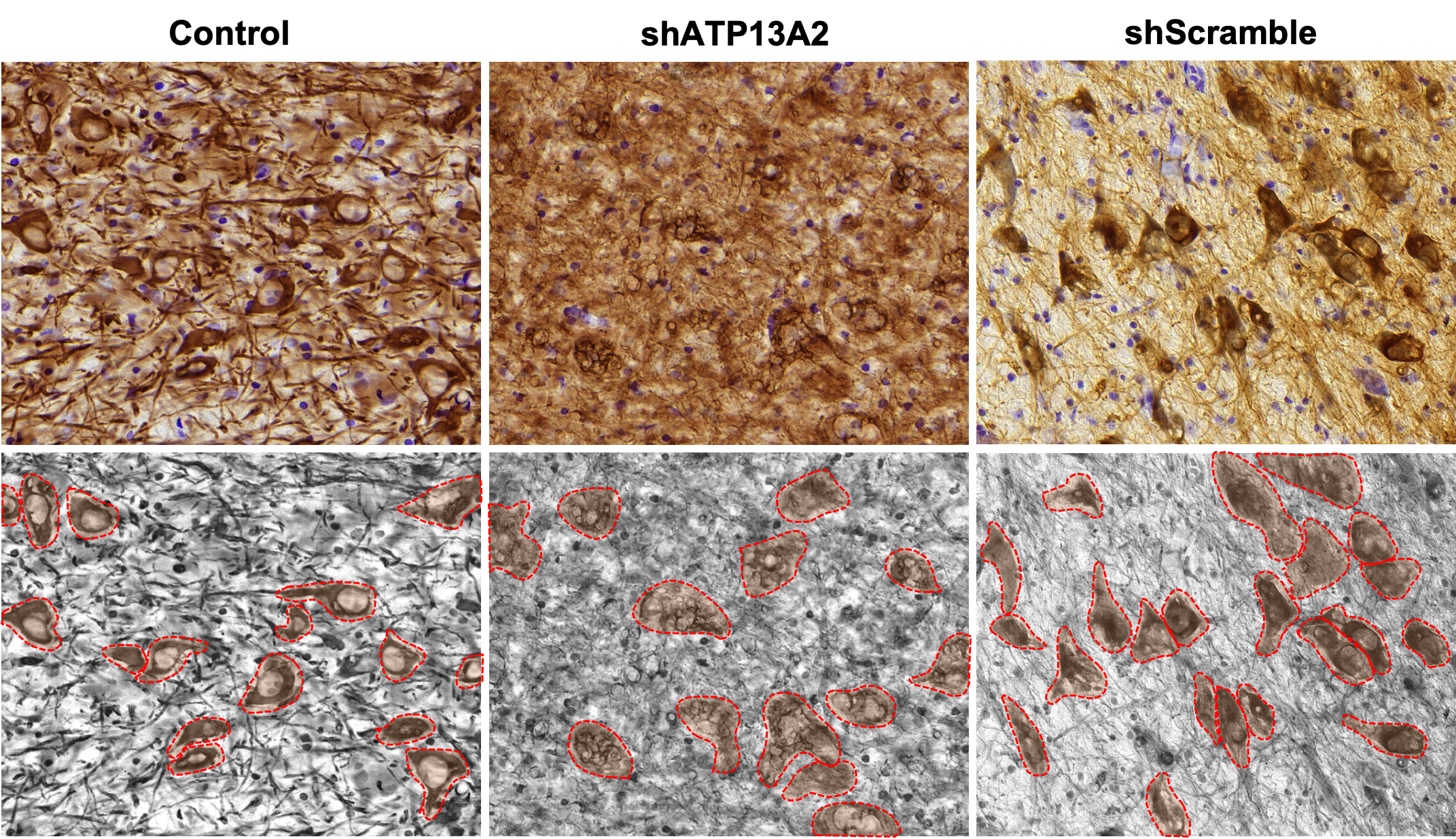


**Supp. Figure 3. Intranigral delivery of a shATP13A2 lentiviral vector alters dopaminergic neurons morphology in macaques.** Representative photomicrographs of TH-immunostained (brown) thionin-counterstained (blue) (*top*), binarized and surrounded by red dotted lines (*bottom*) nigral sections of control (*left*) and shATP13A2-injected (*middle*) and shScramble-injected (*right*) macaque five months post-administration. Scale bar = 20 μm.

**
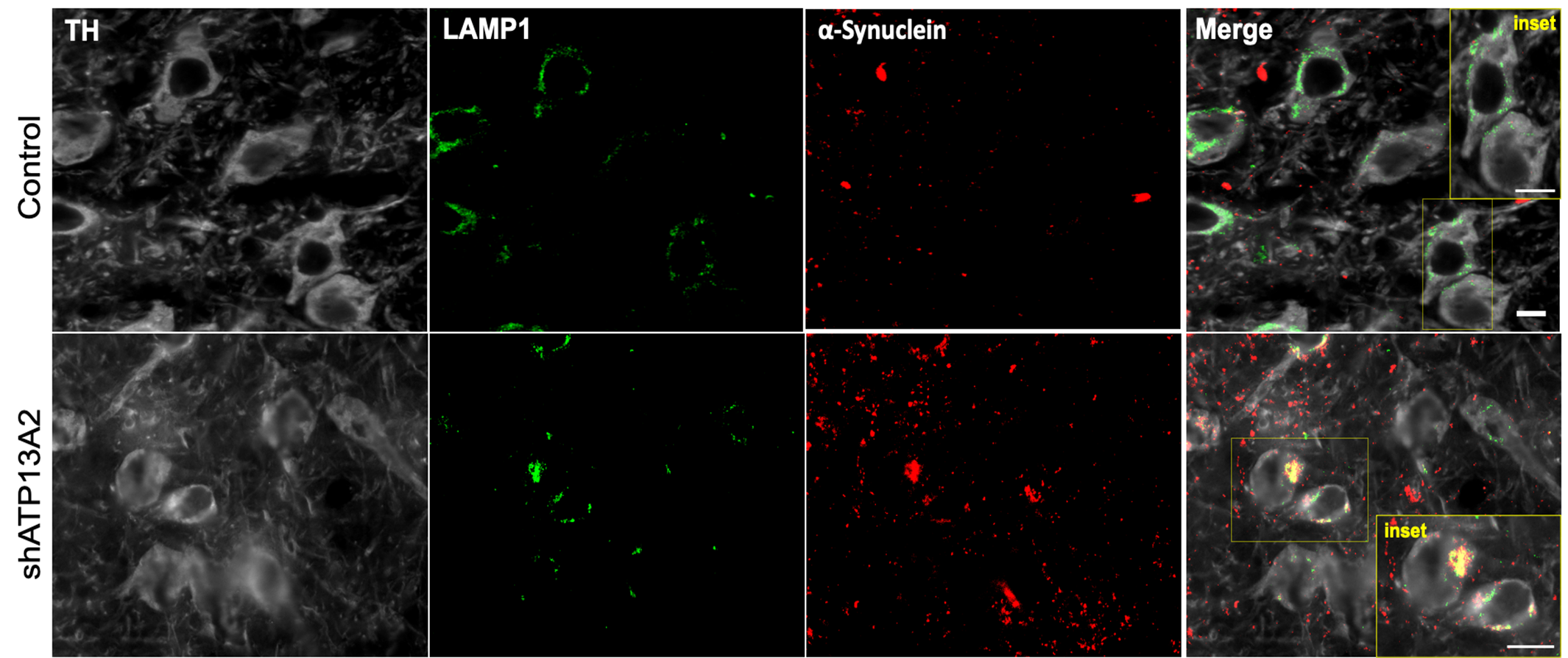
**

**Supp. Figure 4. Intranigral injection of a shATP13A2 lentiviral vector changes the α-syn staining distribution pattern.** Representative confocal micrographs of triple immunostaining for dopaminergic marker tyrosine hydroxylase (TH), lysosomal-related marker (LAMP1) and α-synuclein (syn211 antibody) in the substantia nigra of shATP13A2-injected macaque five months after injection. Scale bar = 10 μm.


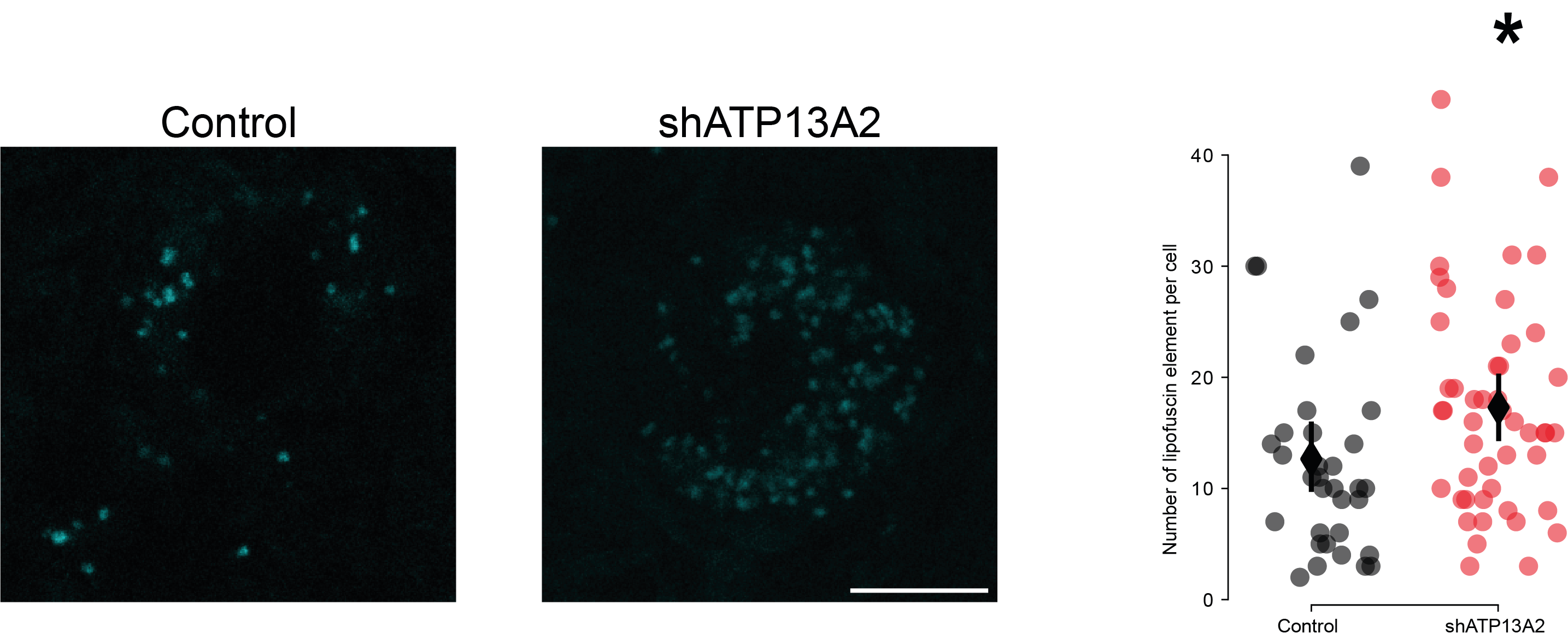


**Supp. Figure 5. Accumulation of lipofuscin in the substantia nigra in shATP13A2 macaques.** Representative images (left) and quantification (right) of lipofuscin element per cell in the substantia nigra in the substantia nigra of the shATP13A2-injected macaques five months post-injection (n = 34-48 cells) (p=0.01455, t=2.222). Scale bar: 10 μm. Comparisons were made using an unpaired Student's t-test. *p-value<0.05 compared to control animals.


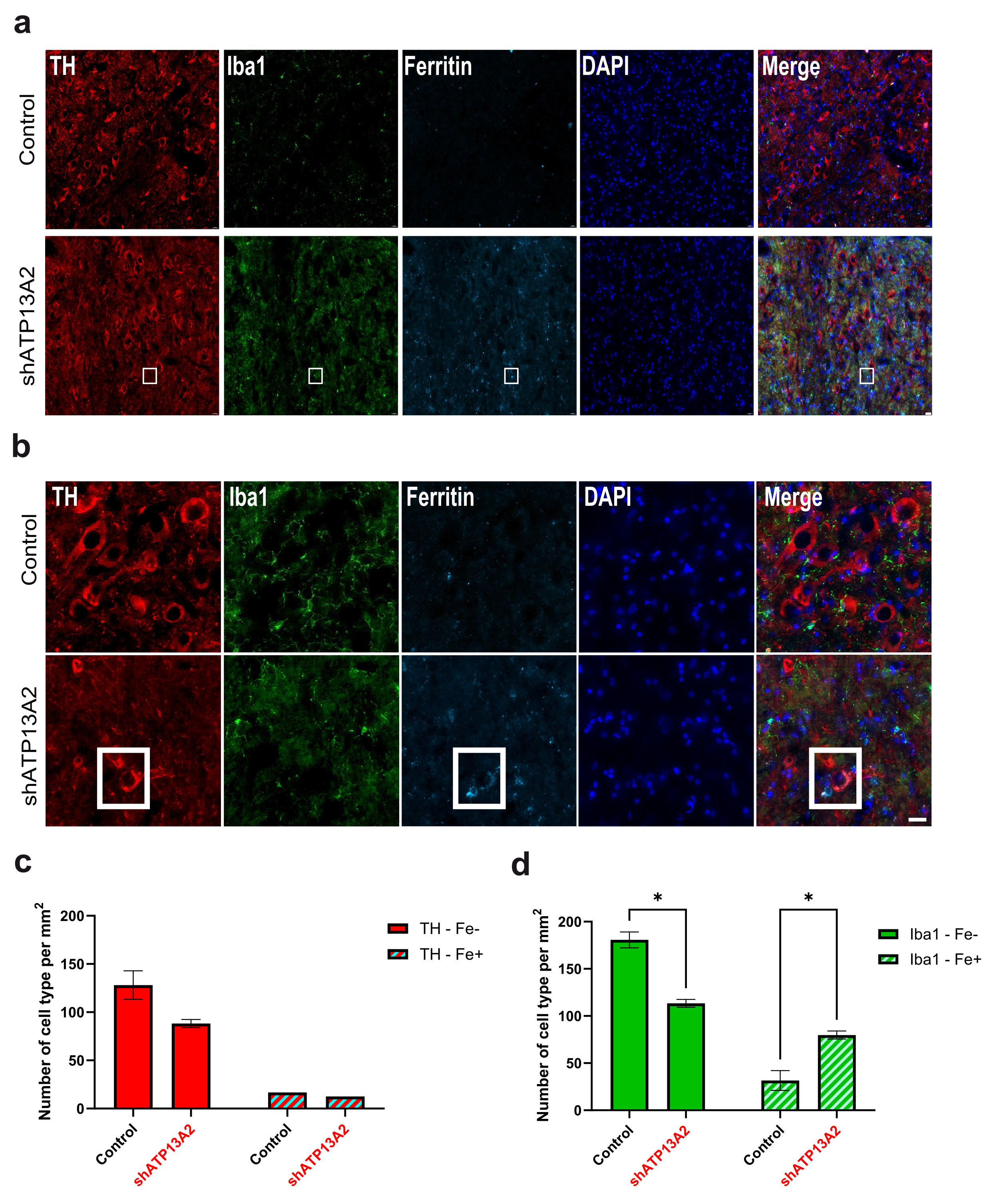


**Supp. Figure 6. Increased ferritin levels in microglia in shATP13A2 macaques.** **(a-b)** Representative confocal micrographs of triple immunostaining for dopaminergic marker tyrosine hydroxylase (TH), microglial marker (Iba1), and ferritin in the substantia nigra of shATP13A2-injected macaque five months post-injection at different magnification (low, **a**; high, **b**). **(c-d)** Quantitative analysis of number of cell types per mm^2^ counted for each experimental group in the substantia nigra performed by counting of TH-positive cells, TH-positive cells colocalizing with ferritin (c), Iba1-positive cells and Iba1-positive cells colocalizing with ferritin (d) on identical ROI of SN confocal images. Statistical analysis was performed using a two-way ANOVA followed by Tukey's post-hoc. *p< 0.05. Scale bar = 10 μm.
